# Genome-wide identification and comprehensive analysis of tubby-like protein gene family in multiple crops

**DOI:** 10.3389/fpls.2022.1093944

**Published:** 2022-12-14

**Authors:** Yafei Zeng, Jianyu Wen, Jinmei Fu, Han Geng, Zhiwu Dan, Weibo Zhao, Wuwu Xu, Wenchao Huang

**Affiliations:** State Key Laboratory of Hybrid Rice, Key Laboratory for Research and Utilization of Heterosis in Indica Rice of Ministry of Agriculture, Engineering Research Center for Plant Biotechnology and Germplasm Utilization of Ministry of Education, College of Life Science, Wuhan University, Wuhan, China

**Keywords:** TLP, gene duplication, cis-elements, expression patterns, seed germination, crops

## Abstract

**Introduction:**

The highly conserved tubby-like proteins (TLPs) play key roles in animal neuronal development and plant growth. The abiotic stress tolerance function of TLPs has been widely explored in plants, however, little is known about comparative studies of TLPs within crops.

**Methods:**

Bioinformatic identification, phylogenetic analysis, Cis-element analysis, expression analysis, Cis-element analysis, expression analysis and so on were explored to analysis the TLP gene family of multiple crops.

**Results:**

In this study, a comprehensive analysis of *TLP* genes were carried out in seven crops to explore whether similar function of TLPs in rice could be achieved in other crops. We identified 20, 9, 14, 11, 12, 35, 14 and 13 *TLP* genes in *Glycine max*, *Hordeum vulgare*, *Sorghum bicolor*, *Arabidopsis thaliana*, *Oryza sativa Japonica*, *Triticum aestivum*, *Setaria italic* and *Zea mays*, respectively. All of them were divided into two groups and ten orthogroups (Ors) based on amino acids. A majority of *TLP* genes had two domains, tubby-like domain and F-box domain, while members of Or5 only had tubby-like domain. In addition, Or5 had more exons and shorter DNA sequences, showing that characteristics of different Ors reflected the differentiated function and feature of *TLP* genes in evolutionary process, and Or5 was the most different from the other Ors. Besides, we recognized 25 *cis*-elements in the promoter of *TLP* genes and explored multiple new regulation pathway of TLPs including light and hormone response. The bioinformatic and transcriptomic analysis implied the stresses induced expression and possible functional redundancy of *TLP* genes. We detected the expression level of 6 *OsTLP* genes at 1 to 6 days after seed germination in rice, and the most obvious changes in these days were appeared in *OsTLP10* and *OsTLP12.*

**Discussion:**

Combined yeast two-hybrid system and pull down assay, we suggested that the *TLP* genes of Or1 may have similar function during seed germination in different species. In general, the results of comprehensive analysis of *TLP* gene family in multiple species provide valuable evolutionary and functional information of *TLP* gene family which are useful for further application and study of *TLP* genes.

## Introduction

The tubby-like proteins (TLPs) were first discovered in obese mouses (Ohlemiller et al., 1995; Kleyn et al., 1996) and mutation of this gene family resulted in obesity, retinal degeneration and hearing loss (Boggon et al., 1999; Ikeda et al., 1999; Ikeda et al., 2002). Gene structure analysis showed that the conserved tubby domain was located in the C-terminal which was important for maintaining neuronal development (Boggon et al., 1999; Ikeda et al., 2002). A typical TLP, containing the conserved tubby domain formed with a β-barrel structure consisting of one α-helix and 12 anti-parallel strands, functions as a bipartite transcription regulator (Carroll et al., 2004). In addition to be identified in mice (Kleyn et al., 1996), human (North et al., 1997) and other animals (Heikenwalder et al., 2001; Figlewicz et al., 2004), this family was also identified in plants such as rice (Kou et al., 2009), Arabidopsis (Lai et al., 2004), wheat (Hong et al., 2015; Li et al., 2018; An et al., 2019), apple (Xu et al., 2016), soybean (Xu et al., 2022) and cassava (Dong et al., 2019). The widespread and high conservation of tubby family in eukaryotes suggested the fundamental function of growth and development in animals and plants (Carroll et al., 2004; Wang et al., 2018; Devane et al., 2022). In addition to the conserved tubby domain in C-terminal, TLPs contained the highly diverse N-terminal sequence in animals, but the conserved F-box at N-terminal in plants (Liu, 2008; Li et al., 2021). The F-box domain was first identified in cyclin F which interacted with the protein S-phase kinase-associated protein 1 (SKP1). F-box proteins and SKP1 were recruited to the Skp1-Cullin1-F-box (SCF) complex *via* interacting with CDC53 (Cullin) proteins to act as part of E3 ubiquitin ligase and participate in protein substrates ubiquitination process in plants (Lai et al., 2004; Horn-Ghetko et al., 2021). In addition to F-box, several typical protein–protein interaction domains of F-box proteins endow protein substrates specificity to the SCF complexes (Lechner et al., 2006). F-box proteins are involved into multiple biological functions including transcriptional regulation, signal transduction and cell cycle transition. In plants, F-box proteins are usually related to abiotic stress, for example, *TaFBA1* in heat tolerance (Li et al., 2018; An et al., 2019), *CaF*-box in response to abiotic stress (Chen et al., 2014), *EST1* in salt tolerance (Liu et al., 2020).

Previous studies have reported that TLPs have a variety of physiological roles in plant, including plant growth and development, disease resistance and abiotic stress responses. In Arabidopsis, 11 TLP members were identified (Bao et al., 2014). *AtTLP2*, *AtTLP6* and *AtTLP7* were preferentially expressed in pollen grains, and *attlp6* and *attlp7* mutant plants showed pollen sterility to some extent (Renak et al., 2012; Xu et al., 2022). AtTLP3 and AtTLP9 interacted with ASK proteins, and *attlp3* and *attlp9* mutant plants exhibited the abscisic acid (ABA) insensitive phenotypes during seed germination (Lai et al., 2004; Bao et al., 2014). The germination time of knock out seeds was a few hours earlier compared with that of the wild-type plants, and the germination frequency of *attlp3/attlp9* was higher than that in single mutant. According to these data, the members of Arabidopsis tubby family may have functional redundancy. In rice, 14 *OsTLPs* were identified and their expression could be induced after pathogen injection (Liu, 2008; Kou et al., 2009). *OsTLP2* had the binding ability to the promoter of *OsWRKY13* to regulate the transcriptional expression of this gene. The *WRKY* family functions as transcriptional activator or repressor in the defense responsive pathway (Cai et al., 2008), thus, TLPs are involved in defense responses. Moreover, the expression level of *CaTLP1* was higher after treatment with dehydration, high concentration NaCl and ABA, showing that the stress responsive function of *CaTLP1* may be related with ABA signaling pathway (Wardhan et al., 2012; Wardhan et al., 2016). The response to abiotic stress of *TLP* family also could be found in other species such as chickpea (Wardhan et al., 2012), soybean (Xu et al., 2022) and *Malus domestica* (Xu et al., 2019).

Based on previous researches in the last decades, multiple *TLP* genes were identified in different species. There are 11 *AtTLP*s in Arabidopsis (Lai et al., 2004), 14 *OsTLP*s in rice (Liu, 2008), 11 *PtTLP*s in poplar (Yang et al., 2008), 4 *TaTLP*s in wheat (Hong et al., 2015), 15 *ZmTLP*s in maize (Dong et al., 2019) and 9 *MdTLP*s in apple (Xu et al., 2016). However, the evolutionary relationship of *TLP* gene family among multiple species in plant were rarely reported. In this study, we identified 117 members in 7 crops including *G.max*, *H.vulgare*, *S.bicolor*, *O.sativa*, *T.aestivum*, *S.italic* and *Z.mays.* Our research focused on the complete gene identification in genome-wide scale, phylogenetic tree construction and orthologous relationships, chromosomal location, whole genome duplication (WGD) or segmental duplication, motif-domain-exon/intron analysis, expression profiles, *cis*-elements analysis and three dimensional structure prediction. Based on systematic analysis of multiple species, these results could provide a more accurate and comprehensive understanding of *TLP* gene family that are useful for molecular function study and breeding application in future.

## Materials and methods

### Plant materials and the germination condition

To verify the expression model of *OsTLP* genes in seeds after germination, the seeds of *Oryza sativa japonica* rice cultivar, Nipponbare, were sterilized with 75% ethanol for 5 min, then soaked by 3% sodium hypochlorite for 30 min and washed by sterile distilled water for five to ten times. In the end, the sterilized seeds were cultivated on 1/2 MS solid medium containing 1% sucrose for different days (Wang et al., 2015).

### Bioinformatic identification and phylogenetic analysis of *TLP* genes

Genome datasets of *Glycine max* (v2.1), *Hordeum vulgare* (IBSCv2), *Sorghum bicolor* (NCBIv3), *Arabidopsis thaliana* (TAIR10), *Oryza sativa Japonica* (IRGSP-1.0), *Triticum aestivum* (IWGSC), *Setaria italic* (v2.0) and *Zea mays* (v4) were obtained from Ensembl Plants (http://plants.ensembl.org/index.html), respectively. The HMM (Hidden Markox Model) profile of the tubby family (PF01167) was download from Pfam (http://pfam.xfam.org/). Then the members of *TLP* family were obtained by HMMER 3.2.1 software by the command ‘hmmsearch PF01167.hmm species-protein.fasta > species-TLP.out’ with default parameters (E-value of ≤0.01) (Finn et al., 2016; Dong et al., 2019) and BLASTP method with a cut-off E-value of e^−5^ (Altschul et al., 1997; Kong et al., 2019). All candidate sequences were detected by SMART (http://smart.embl-heidelberg.de/) and Pfam (http://pfam.xfam.org/search/sequence) to filter out the incomplete protein sequences and duplicated transcripts.

The protein sequences were submitted to ClustalW v2.1, then the phylogeny tree was produced using MEGA 6.0 software by the neighbor-joining method with 1,000 bootstrap replicates (Tamura et al., 2013). Names of putative *TLP* genes were obtained according to the value of assigned principally based on E-value of BLASTP.

### Chromosomal location, conserved motifs, domains and gene structure

The Chromosomal location, motifs, domains and gene structure were visualized using TBtools v0.665. The genome annotation files were also downloaded from Ensembl Plants. The messages of gene location and exon/intron were obtained in these files. The 20 conserved motifs were obtained from MEME Suite 5.0.2 (http://meme-suite.org/tools/meme). Then, the identified motifs were annotated by Pfam and InterProScan (http://www.ebi.ac.uk/interpro/search/sequence-search) databases. The protein sequences of *TLP* family were submitted to NCBI for recognizing the conserved domains.

### Gene duplication events and Ors identification

Gene duplication events of the *TLP* gene family in intra-species and among species were analyzed and classified by MCScanX software. MCScanX software was commonly used to calculate the collinearity of gene-pairs. In general, if the gene-pairs within two segmental regions had collinearity, the WGD or segmental duplication event was defined. If the distance between duplicate genes was less than 20 gene loci, we named the gene-pairs as tandem duplications or proximal duplications. Then the Ka (Nonsynonymous substitution rate), Ks (Synonymous substitution rate), Ka/Ks (Selective strength) of gene-pairs were analyzed by DnaSP 5.0. In general, if Ka/Ks >1, there was positive selection between gene-pairs. If Ka/Ks = 1, there was Neutral selection between gene-pairs. If 0 < Ka/Ks < 1, there was Negative selection between gene-pairs. Besides, the divergence time was calculated as T = Ks/(2 × 6.5 × 10^−9^) × 10^−6^ Mya.

The Ors were obtained using the OrthoFinder v2.2.6 software. All protein sequences were sorted by species in different files, this analysis was conducted by the command “orthofinder – d Exampledata – s diamond”. Then the results were analysis according to published methods.

### *Cis*-element analysis and expression analysis of *TLP* genes

For *cis*-element analysis, using genome sequence and genome annotation, the 2000 bp promoter sequences of all *TLP* genes in this study were extracted using TBtools v0.665 software. Then these sequences were submitted to the *cis*-element analysis website: PLANTCARE (http://bioinformatics.psb.ugent.be/webtools/plantcare/html) and selected the Search for CARE function, and the analysis results would be obtained (Lescot et al., 2002). The summary file obtained total information of *cis*-element, and we retained all the significative information and sites according to the last column function annotation. Finally, the information was visualized by ‘Simple Bio Sequence Viewer’ function in TBtool v0.665 (Chen et al., 2020).

In this study, we analyzed the RNA-seq data of *TLP*s in three major food crops (rice, wheat and maize). The expression data of rice and maize at different periods and organizations were obtained from rice and maize eFP Browser. The expression data of wheat at different periods and organizations were obtained from expVIP website. There were 15, 15 and 79 tissue types of rice, wheat and maize. In order to measure the expression of *OsTLPs* in environment stress, the transcriptome data was also downloaded from rice eFP Browser. Four heatmaps were drawn using TBtools v0.665 software.

### Predicted the protein structure

Three dimensional structure of OsTLP proteins were predicted by SWISS MODEL. The amino acid sequences were submitted to the SWISS MODEL website. The template 1S31.1.A was selected for OsTLP proteins. The sequence identities between samples and template were over 40%. The predicted protein structure was analyzed and highlight *via* the Swiss PdbViewer v4.1.0.

### RNA extraction and qRT–PCR

The total RNA was extracted from seeds using TRIzol according to the manufacturer’s instruction book. Then the DNase I was employed to remove genome DNA. Then the total RNA was dissolved using RNase-free water.

The cDNA was obtained *via* reverse transcription reagent (Invitrogen) according to the manufacturer’s instructions. The qRT-PCR was performed using the LightCycler 480 system (Roche) and the cycling conditions for qRT-PCR were as follows: firstly, 5 min at 95 °C; secondly, 10 seconds of denaturation at 95 °C, 20 seconds of annealing at 58 °C, and 20 seconds of extension at 72 °C, for 40 cycles. The actin gene was selected as an internal control. The primers were listed in **Supplementary Table 9**.

### Validation with the yeast two-hybrid system

The *OsTLP10* and *OsTLP12* were cloned into pGADT7 and the sequence of *OSK20* was cloned into pGBKT7. Then these vectors were cotransformed into the AH109 yeast strain according to different groups. The transformed cells were grown on SD/-Leu/-Trp plates and then grown on SD/-Leu/-Trp/-His/-Ade plates at 30°C for 3–5 days. The primers were listed in **Supplementary Table 9**.

### Pull down assay

The *OsTLP10* was cloned into pGEX-6p-1 vector which contains a GST tag. The *OSK20* was inserted into pET-28a vector which has a His tag. The recombinant plasmids were transformed into the Escherichia coli strain BL21, respectively. Then the proteins were induced and expressed by IPTG at 20°C overnight. Proteins were purified using AKTAprime Plus (GE), separated by 10% SDS-PAGE gels, and tested by the antibodies.

The purified recombinant proteins (GST tag, GST-OsTLP10 and His-OSK20) were dialyzed against phosphate-buffered saline (PBS; 137 mM NaCl, 2.7 mM KCl, 10 mM Na_2_HPO_4_, and 2 mM KH_2_PO_4_) overnight and then quantified using the bicinchoninic acid (BCA) method. The recombinant His-OSK20 proteins were incubated with GST and GST-OsTLP10 protein for 6 h on ice and then washed three times with five volumes of PBS. Then the mixture was separated by 10% SDS-PAGE. The products were transferred onto a polyvinylidene fluoride (PVDF) membrane (Bio-Rad) and investigated with antibodies of GST and His. The primers were listed in **Supplementary Table 9**.

### Expression profiles of *TLP* genes in different species

The expression data of *TaTLP* genes were downloaded from the expVIP website (Borrill et al., 2016) and the expression of *OsTLP* genes and *ZmTLP* genes were downloaded from eFP Browser (http://bar.utoronto.ca/transcriptomics/efp_rice/cgi-bin/efpWeb.cgi and http://bar.utoronto.ca/efp_maize/cgi-bin/efpWeb.cgi). In order to verify the response of *OsTLP* genes to abiotic stresses, the expression data of *OsTLP* genes under cold, drought, salt environments were also downloaded from rice eFP Browser. These data were exhibited in **Supplementary Table 7** and **Supplementary Table 8**.

## Results

### Phylogenetic analysis and classification of *TLP* genes in multiple crops

**Figure 1:**
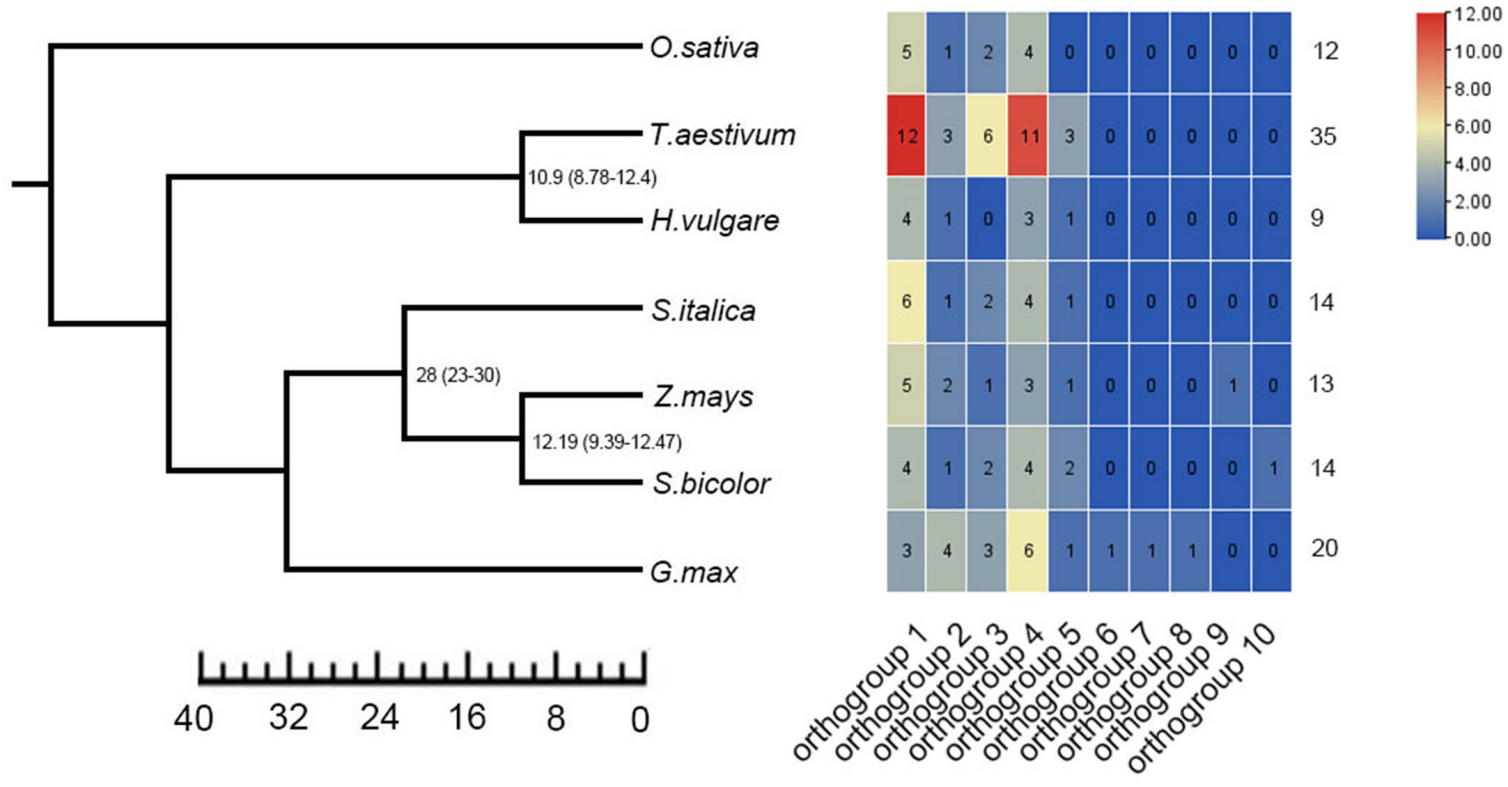
Identification of *TLP* genes in seven crops. Ten orthogroups and number of *TLP* genes in different species were shown by heat map. The value was represented in colors.

**Figure 2:**
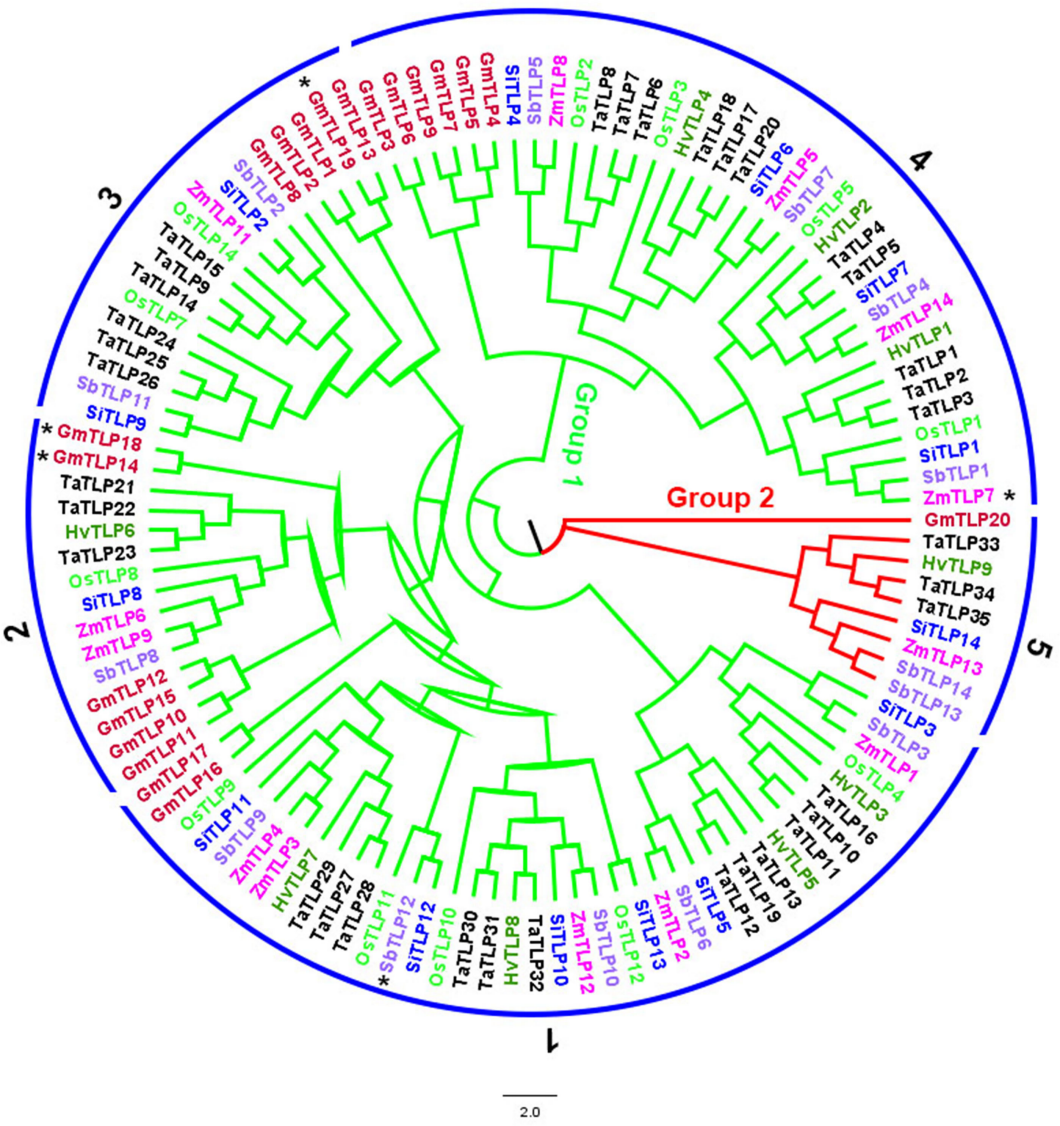
The phylogenetic classification of TLP proteins from *G.max*, *H. vulgare*, *S.bicolor*, *O.sativa*, *T.aestivum*, *S.italic* and *Z.mays*. Different colors represent different crops. Ors were marked by numbers and GmTLP14*, GmTLP18*, GmTLP19* ZmTLP7* and SbTLP12* were belonged to Or6, Or7, Or8, Or9, Or10.

To identify the copy number variation of *TLP* genes, we obtained a total of 117 members of *TLP* genes from seven crops, including 20 in *G.max*, 9 in *H.vulgare*, 14 in *S.bicolor*, 12 in *O.sativa*, 35 in *T.aestivum*, 14 in *S.italic* and 13 in *Z.mays* (**Figure 1**; **Supplementary Table 1**). We also identified 11 *AtTLP* genes in *A.thaliana*, which was consistent with previous job, illustrating the reliable of *TLP* genes that we identified in these crops. Previous jobs identified 14 and 15 *TLP* genes in rice and maize, respectively, whereas we found that *OsTLP6*, *ZmTLP10* and *ZmTLP15* could not form the entire tubby domain (**Supplementary Table 2**), and we did not find the *OsTLP13* in rice. The TLPs ranged from 33.1 (OsTLP14) to 60.2 (SiTLP10) kDa in relative molecular weight and 9.01 (GmTLP15) to 10.15 (ZmTLP13) in isoelectric point (**Supplementary Table 3**). To explore the evolutionary relationships of the *TLP* genes in these crops, the phylogenetic tree and Ors were employed (**Figures 1**, **2**). According to the amino acids, these genes were divided into two groups which consisted 10 Ors, and the size of Or1 and Or4 were bigger than others (**Figure 2**). In addition, all seven species could be found in Or1, Or2 and Or4, while Or6, Or7, Or8, Or9 and Or10 were lineage-specific which only had one species (**Supplementary Table 4**). According to these results, we drew a conclusion that the Or1 and Or4 were more conservative compared with the other Ors. Moreover, the members in *G.max* were classified into eight Or groups, while there were only four in *O.sativa*. These results illuminated that the members of *TLP* gene family had lineage-specific expansions and happened homologous gene loss/gain in the process of plant evolution.

### Chromosomal distribution and gene duplication analysis

**Figure 3:**
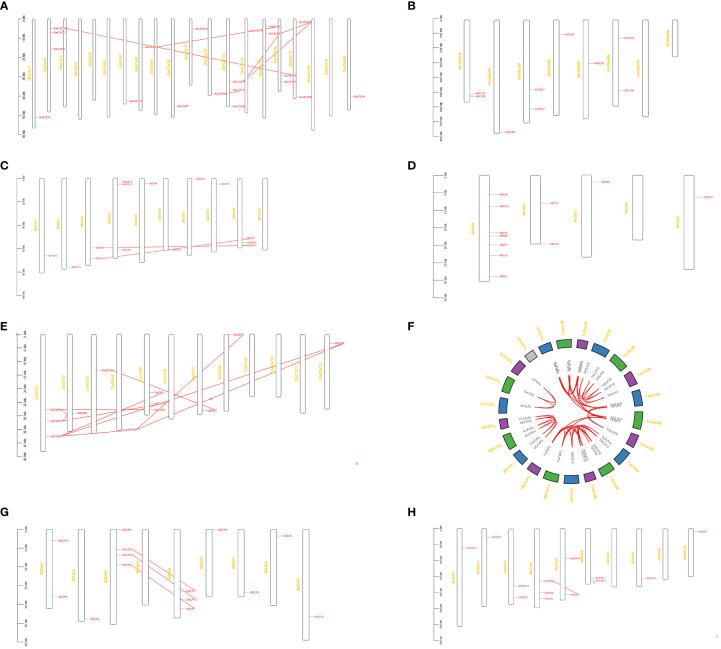
The chromosome distribution and duplication analysis of *TLP* genes in eight species. *G.max* **(A)**, *H vulgare* **(B)**, *S.bicolor* **(C)**, *A thaliana* **(D)**, *O.sativa* **(E)**, *T.aestivum* **(F)**, *S.italic* **(G)**, *and Z.mays* **(H)**. The red lines showed WGD or segmental duplication.

The distribution of *TLP* genes was identified by mapping their sequences to the chromosome to reveal the expansion mechanism of paralogues in *TLP* genes. We inquired the gene location in the genome of seven crops and identified duplication modes of each crop. As shown in **Figure 3**, there were three *OsTLP* genes on Chr1 and Chr5, one *OsTLP* on Chr2, Chr3, Chr4, Chr7, Chr8 and Chr12 in rice, while no *OsTLP* gene was identified in other Chrs (**Figure 3E**). A few *TLP* genes were localized at the proximal regions of relevant chromosomes where they were unstable and more easily to lose due to chromosome replication, such as *GmTLP20*, *SbTLP5* and *OsTLP2* (**Figures 3A, C, E**). Moreover, we found the whole genome duplication (WGD) or segmental duplication events in *G.max*, *S.bicolor*, *O.sativa*, *T.aestivum*, *S.italic*, and *Z.mays*, but no WGD or segmental duplication event was happened in *H. vulgare* and *A. thaliana* (**Figures 3B, D**). Besides, tandem duplication and proximal duplication events did not appear in our results (**Figure 3**). Most of gene-pairs were located in the same Or, such as *OsTLP9-OsTLP12 andGmTLP15-GmTLP12*, suggesting that gene expansions primarily occured in Or internally. Interestingly, we also found some gene-pairs from different Ors, such as *GmTLP1-GmTLP19* and *SbTLP9-SbTLP12*, indicating that Or6-Or10 may be separated from conserved Or groups. Finally, Ka/Ks ratios of gene-pairs were calculated and all of them were less than 1, so these events were purifying selection according to the neutral theory. The Divergence times of the same specie were similar, but it had a big difference in different species (**Supplementary Table 5**).

**Figure 4:**
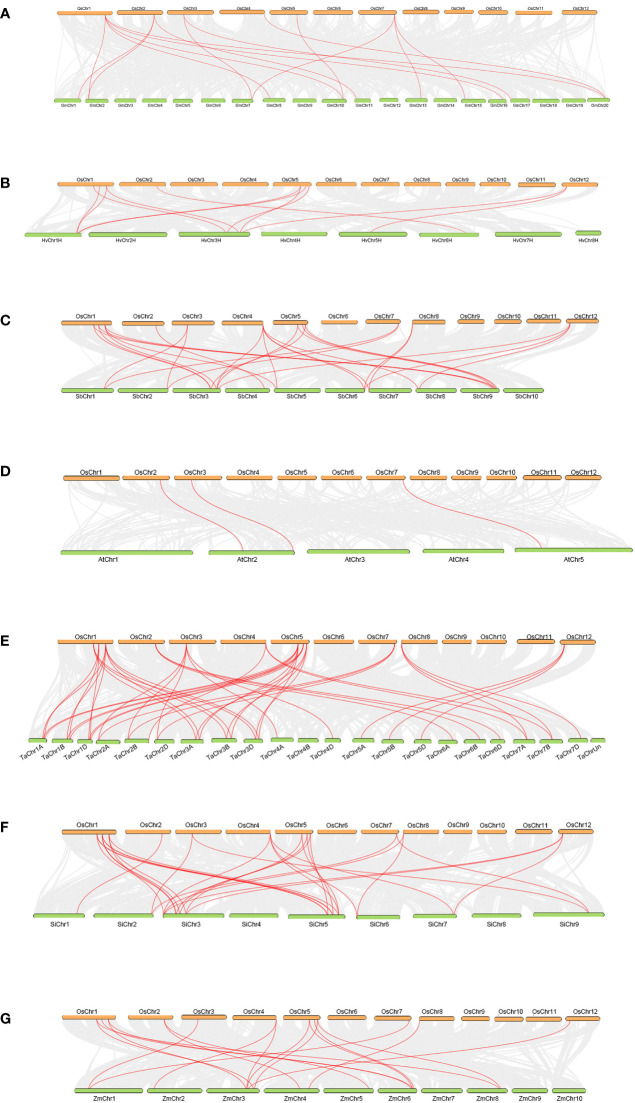
Collinearity between OsTLPs and TLPs from other seven species. O.sativa vs G.max **(A)**, O.sativa vs *H* vulgare **(B)**, O.sativa vs S.bicolor **(C)**, O.sativa vs **(A)** thaliana **(D)**, O.sativa vs T.aestivum **(E)**, O.sativa vs S.italic **(F)**, and O.sativa vs Z.mays **(G)**. The grey lines represent background which indicated the collinear blocks within the genomes of O. sativa and other species, TLP pairs were marked by red lines.

**Figure 5:**
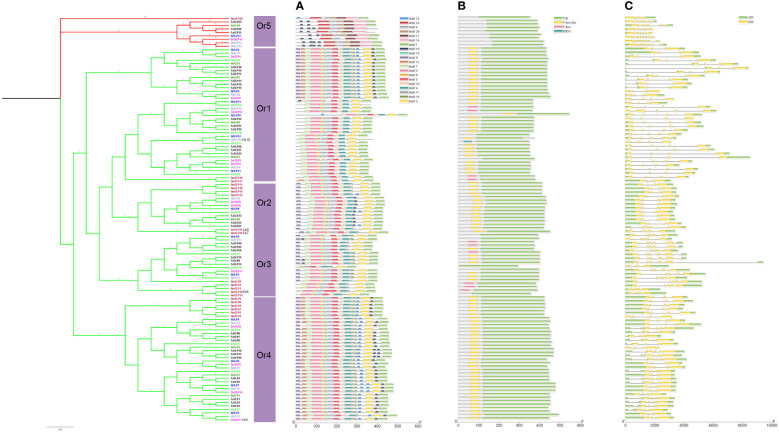
The phylogenetic tree, conserved motif, domain, exon/intron structure compositions of the *TLP* genes in seven species. Phylogeny tree of TLP proteins from seven species. Different colors of branches represented different groups. The different species were showed by different colors. **(A)** Conserved motif of TLP proteins. **(B)** Domain analysis of TLP proteins. **(C)** The structure of *TLPs* contained CDS and UTR.

There were 8, 12, 12, 12 and 12 *OsTLPs* that had collinearity with the *TLP*s of five gramineae crops (monocots), *H.vulgare*, *S.bicolor*, *T.aestivum*, *S.italic* and *Z.mays*. Meanwhile, there were 6 and 3 in two dicots, *G.max*, and *A.thaliana*, respectively (**Figure 4**; **Supplementary Table 6**). Only *OsTLP8* had collinearity with the *TLPs* from all of the seven species (**Figure 5**; **Supplementary Table 6**). Obviously, there was a closer collinear relationship within the gramineae crops.

These results above indicated that duplication events were important for *TLP* genes expansion which happened in different periods (**Supplementary Table 5**). Besides, the closer collinear relationship within the gramineae crops indicated that the relationship of *TLP* genes was more closely in the interior of monocots.

### Conserved motifs, functional domains and gene structure analysis

To explore the functional properties of TLPs, we analyzed the motifs and domains in these species. We identified 20 conserved motifs using MEME website (**Supplementary Figure 1**). The motifs in **Figure 5A** showed that the genes in the same Or had the similar motifs, indicating that they had high conservation in the same Or. Notably, the motif 1 that almost exits in all members of *TLPs* gene was located in the C-terminal of tubby domain, implying that the amino acids of this part were very conserved among different Ors and the conserved function of *TLP* proteins may be determined by this motif region. Interestingly, we found that multiple motifs of Or5 did not exist in other Ors, suggesting that there may be some unidentified functions in Or5 (**Figure 5A**).

Next, we analyzed the domains of TLPs, and the data showed that F-box-like domain appeared more often than any other domains except tubby domain (**Figure 5B**). Previous jobs showed that the F-box domain is related with plant resistance response to environmental stress (Lechner et al., 2006; Chen et al., 2014). In addition, the group 2 did not have the F-box in N-terminal, which was more similar to the *TLP* gene family of animals (**Figure 5B**) Thus, we speculated that the members of group 1 may be more adaptable to environment compared with group 2.

Comparing with the regularity of motifs and domains, the gene structures of *TLP* family were different between different Ors. The group 2 of the *TLP* genes which was only concluded Or5 had more exons and shorter DNA sequences compared with others (**Figure 5C**). Thus, the *TLP* genes had been differentiated into different structures and functions.

### The *cis*-elements in the *TLP* promoter

**Figure 6:**
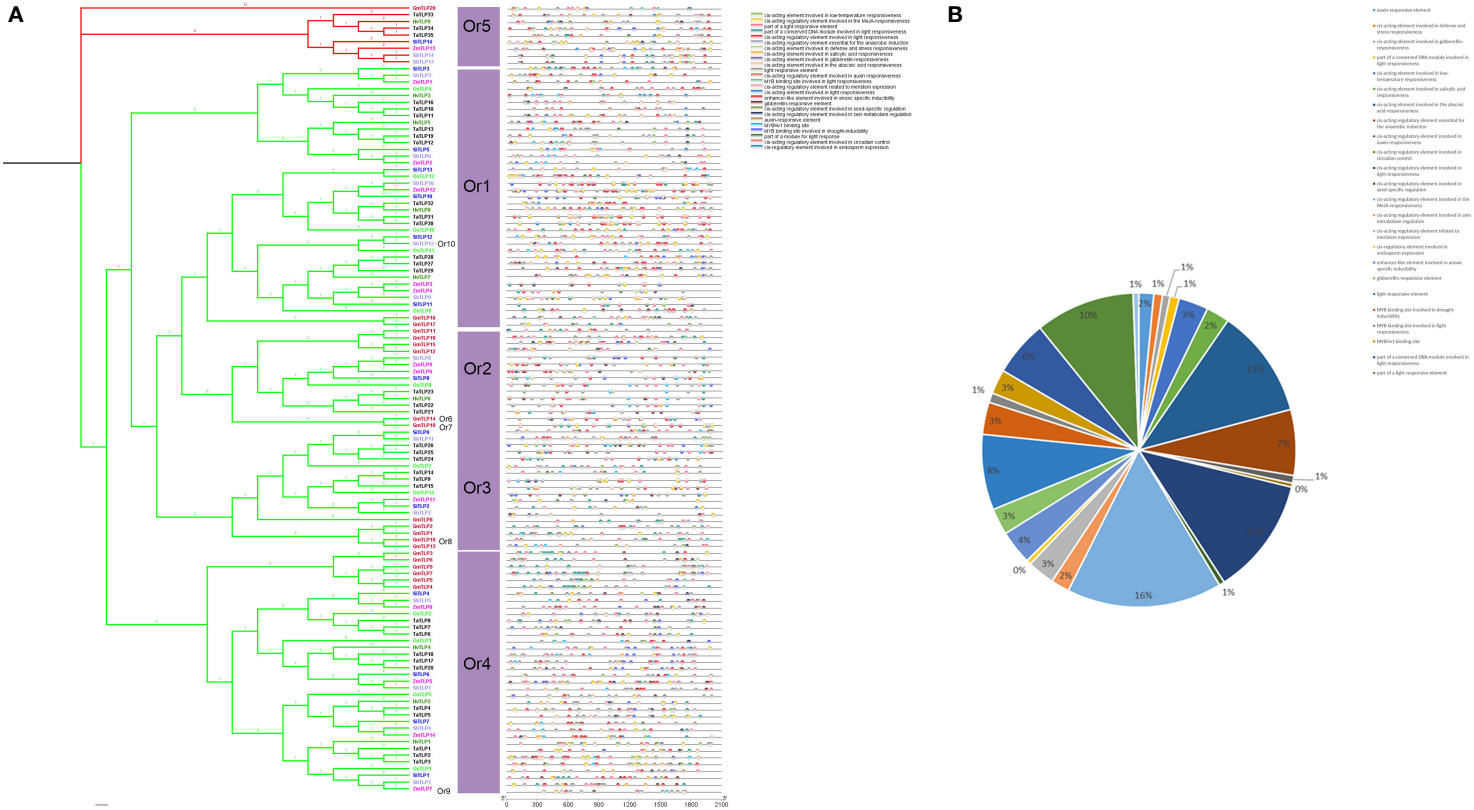
Cis-Acting elements in the promoter of *TLP* genes. **(A)** Cis-Acting elements of *TLP* genes were marked by different color box **(B)** The percent of different promoter element were shown by pie chart.

The transcription of genes was determined by the promoter, and the *cis*-elements could provide multiple information for gene function. Thus, we identified 25 cis-elements in *TLP* genes which were mostly involved in abiotic stress, such as low temperature responsiveness, anoxic induce and abscisic acid responsiveness (**Figure 6**). Notably, we also found a lot of light response elements and hormone responsive element. Moreover, there were many binding sites of MYB proteins which had been proved to participate in cold, drought and plant development as the transcription factor (Cominelli and Tonelli, 2009; Dubos et al., 2010; Yan et al., 2021). However, the regulation pathway between MYB and *TLPs* had not been reported. Taken together, we inferred that *TLP* genes participated in light responsiveness, hormone pathway and plant development.

### The expression patterns of *TLP* genes in three major food crops

**Figure 7:**
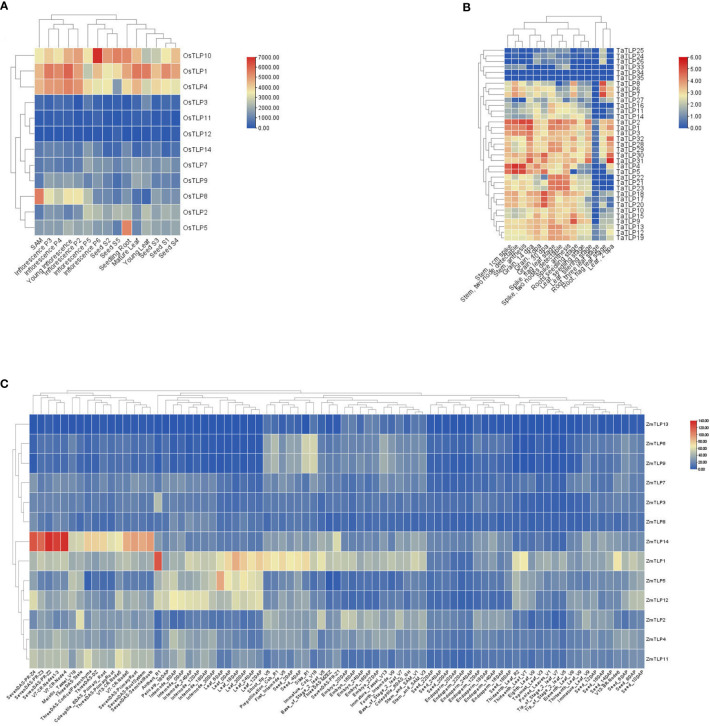
Expression profiles of *TLP* in three cereal crops. *O.sativa* **(A)**, *T.aestivum* **(B)**, and *Z.mays* **(C)**. The red/blue indicated high/low level of *TLP* transcript expression.

In order to confirm whether *TLP* genes are involved in the abiotic stress and plant development of the above results, we identified the expression patterns of *TLP* genes in three major food crops, rice, maize and wheat (**Supplementary Table 7**). According to the expression profile in **Figure 7**, the expression pattern of *TLP* genes could be classified into two groups, some genes had higher expression levels anywhere, such as *OsTLP10*, *OsTLP1*, *OsTLP4*, *TaTLP1*, *TaTLP2* and *TaTLP3*, some *TLP*s genes were hard to detect, such as *OsTLP3*, *OsTLP11*, *OsTLP12*, *TaTLP33*, *TaTLP34* and *TaTLP35* (**Figure 7**). We noted that the expression levels of *TLP* genes were higher in inflorescence and grain than that in root, seed and leaf.

**Figure 8:**
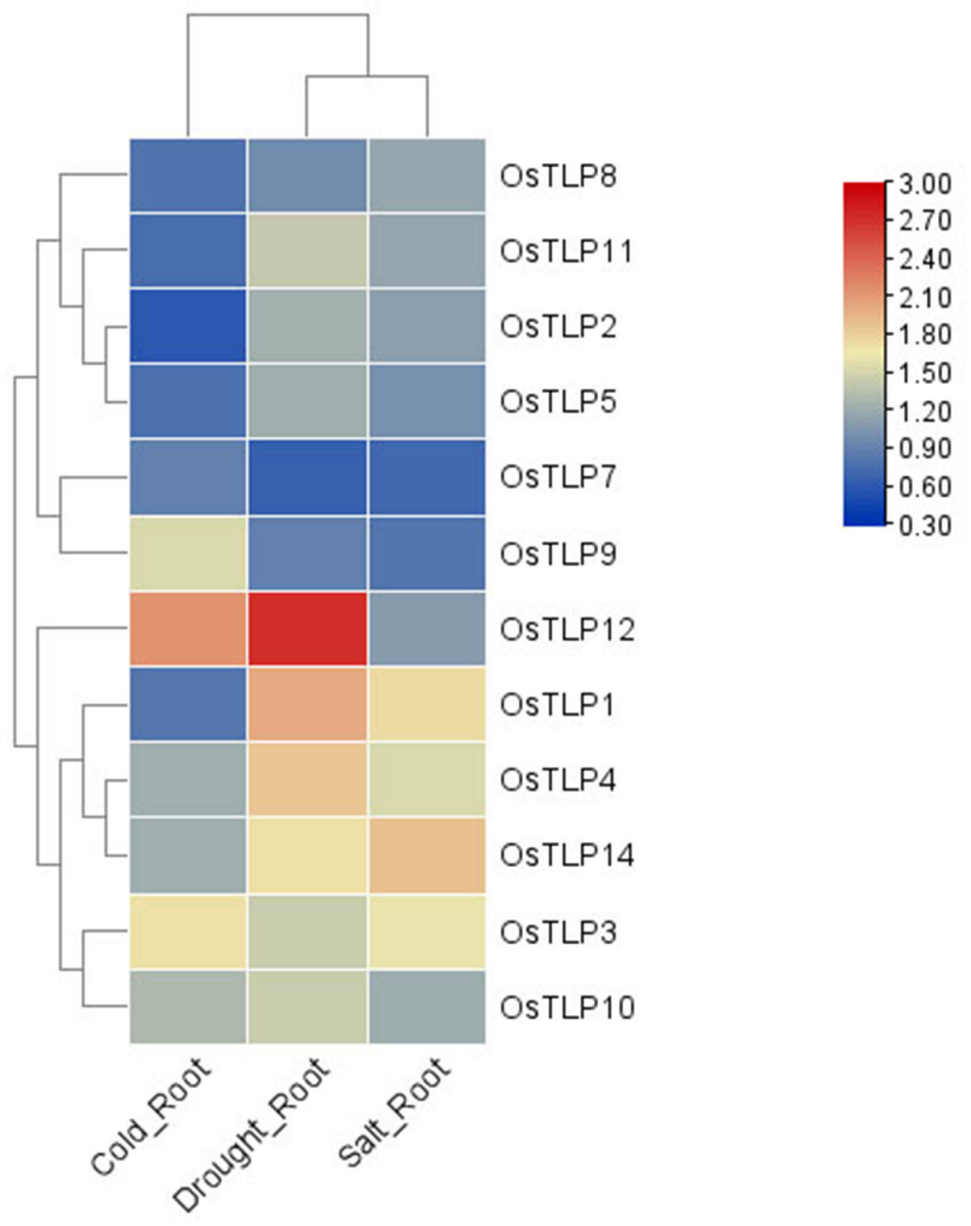
Heatmap depicts expression change folds of *TLP* genes under different conditions. The abiotic stresses included cold, drought and salt conditions. The different change folds were represented by different colors.

Previous jobs have proved that the *TLP* family has important roles in stress response in plant. Thus, we measured the expression level of *OsTLPs* under cold, salt, and drought conditions. As expected, the expression levels of all genes were upregulated in these conditions (**Figure 8**; **Supplementary Table 8**). Indeed, the genes *OsTLP10*, *OsTLP1* and *OsTLP4* with high expression levels were largely upregulated in response to stresses, while the *OsTLP12*, *OsTLP14* and *OsTLP3* genes with low expression levels were also highly upregulated in response to stresses. These results confirmed that partial of *TLP* genes could be induced by different environment stresses.

### Three dimensional structure of OsTLPs

**Figure 9:**
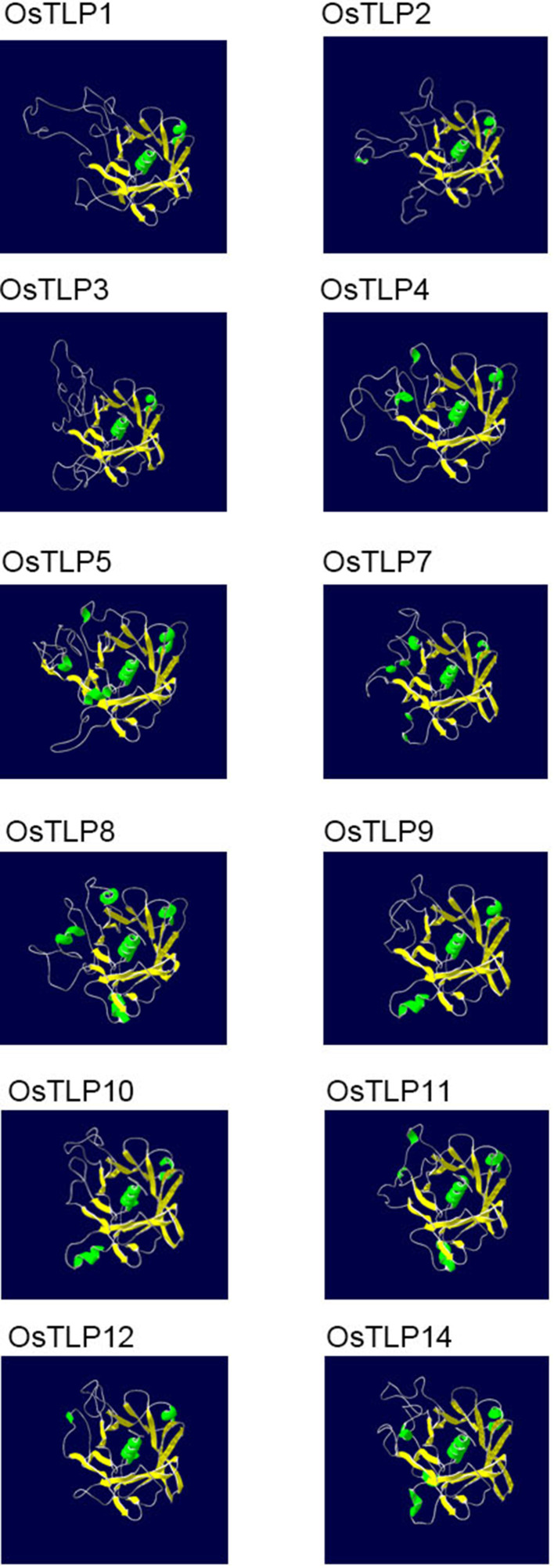
The three dimensional structure of OsTLPs predicted by SWISS Model. The α helixes and β-folds are shown in green and yellow, respectively.

The function of protein was also determined by their structures, thus we obtained the structure of OsTLPs from swiss modle website. Previous jobs suggested that a typical tubby was a β-barrel shape which contained one α-helix and 12 anti-parallel strands. As shown in **Figure 9**, all of the OsTLPs had one central α-helix and 14-17 anti-parallel strands. The Or1 members, *OsTLP4*, *OsTLP9*, *OsTLP10*, *OsTLP11* and *OsTLP12*, had 14 anti-parallel strands. Only one OsTLP, OsTLP8, which was the only member of Or2, had 15 anti-parallel strands. Together, we found that the three dimensional structure of all OsTLPs were similar and they were more conserved in the same Or.

### The expression pattern of *OsTLPs* in seed germination

**Figure 10:**
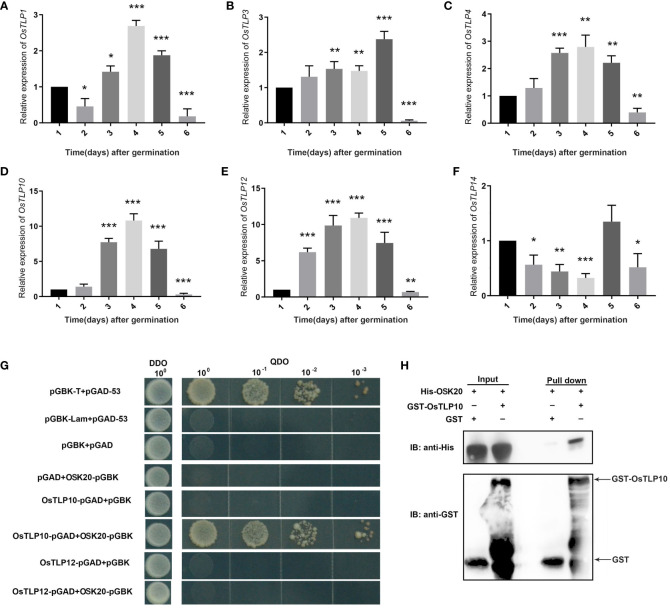
Expression levels and interaction ability of *OsTLPs.* **(A-F)** Relative expression of *OsTLPs* in seeds after germination. Data represent mean ± SD from three biological replicates (Student’s t test, *P < 0.05, **P < 0.01, ***P < 0.001). **(G)** Interaction of OsTLP10 and OsTLP12 with OsK20 by yeast two-hybrid analysis. The full-length OsTLP10 and OsTLP12 were fused with GAL4 activation domain and OsK20 protein was fused with GAL4 DNA binding-domain, respectively. pGBK-T, pGAD-53 were used as positive control and pGBK-lam, pGAD-53 were chosen as negative control. **(H)** Pull down assay was employed to show the interaction relationship between OsTLP10 and OsK20. IB, immunoblotting. The fusion proteins His-OsK20 and GST-OsTLP10 were detected by anti-His and anti-GST antibodies, respectively.

The expression pattern of *OsTLPs* above implied that the *OsTLP1*, *OsTLP3*, *OsTLP4*, *OsTLP10*, *OsTLP12* and *OsTLP14* were related with environment stresses. Based on previous researches, *TLP* gene family also took part in seed germination (Li et al., 2021), thus we identified the expression levels of these six genes at 1 to 6 days after seed germination in rice. All the six genes could be detected in the seed germination period, and the highest expression level presented at the third or fourth day (**Figure 10**). Especially, we found that the most obvious changes appeared in *OsTLP10* and *OsTLP12.* Interestingly, *OsTLP10* and *OsTLP12* were the closest relatives in the phylogenetic evolution tree (**Figure 2**). Previous jobs showed that TLPs interacted with SKP1-like proteins which also participated in seed germination. Thus, we observed the interactions among the OsTLPs and OSKs (*SKP1*-like genes in rice). Previous study reported that *OSK1, OSK8, OSK11, OSK20, OSK23, OSK24, OSK29*, and *OSK31* could be detect in seed, but only OSK1 and OSK20 could interact with F-box genes. Furthermore, *OSK1* was constitutive expressed in multiple tissues while *OSK20* was only highly expressed in endosperm (Kahloul et al., 2013). This implied that *OsK20* may play key roles in seed germination. The yeast two-hybrid was employed to verify the interaction between OsTLP10, OsTLP12 and OSK20. As shown in **Figure 10G**, the interaction was observed between OsTLP10 and OSK20, but no positive strains could be found between OsTLP12 and OSK20. Moreover, the interaction of OsTLP10 and OSK20 was further confirmed by pull down assay. Based on the bioinformatic analysis and molecular verification, we suggested that OsTLP10 and OSK20 may functioned together to regulate seed germination.

## Discussion

Previous jobs proved that the TLPs play a great role in plant development and response to multiple environment stresses in Arabidopsis and cassava (Bao et al., 2014; Dong et al., 2019). In this work, we first identified the members of *TLP* genes in multiple crops, which included the species not yet being reported, *H.vulgare*, *S.bicolor*, and *S.italic*. We identified 20 *TLPs* in *G.max*, 9 in *H. vulgare*, 14 in *S.bicolor*, 12 in *O.sativa*, 35 in *T.aestivum*, 14 in *S.italic* and 13 in *Z.mays*. We found that most of species had 10-15 *TLPs*, whereas there was 35 *TaTLPs* in *T.aestivum*, may be owing to the hexaploid of wheat. By comparing the *TLP* genes among various species, we found that there was no direct relationship between the number of genes and genome size. For example, there were 20 and 13 members in *G. max* (genome size: 1100 Mbp) and Z.mays (genome size: 2300 Mbp). Besides, *H. vulgare* only had 9 *TLP* genes, which was the fewest in this study, while the genome size reached 5.1Gb. Furthermore, the specific WGD event which happened in *Z. mays* did not increase the number of ZmTLPs, indicating no direct relevance between WGDs and the number of genes.

According to the phylogenetic evolution tree and Ors, the 117 members can be classed two groups and 10 Ors, and the Or1 and Or4 were more conservative compared with the other Ors. The members in conserved Ors usually showed higher expression levels, which was obvious in rice and wheat. The *TLP* gene family in same Ors exhibited similar motifs, domains, gene structure, *cis*-elements and expression pattern, thus we speculated that the expression of partial *TLP* genes were enough for plant growth and development in the normal growing environment, and *TLP* genes had function redundance and could be induced in changing environment. For Or5, which was the only members of group2, had multiple motifs that did not exist in other Ors, more exons and shorter DNA sequences than other Ors, and did not have the F-box in N-terminal, indicating that the *TLP* genes in Or5 may have completely different function, which need to be further explored.

Previous jobs of TLPs concentrated on plant development and plant response to environment stress (Lai et al., 2004; Bao et al., 2014; Wardhan et al., 2016). According to the analysis of *cis*-elements in the *TLP* promoter, we obtained additional regulation information. In addition to low temperature and drought, TLPs were also involved in the response of light and hormone, such as auxin, salicylic acid and gibberellin. Interestingly, we also identified several transcriptional factors involved in TLPs regulatory pathway, such as the *MYB* family, which are the significant transcription factors present in all eukaryote and involved in the cell cycle, responses to biotic and abiotic stresses and plant development (Cominelli and Tonelli, 2009; Dubos et al., 2010; Yan et al., 2021). The typical tubby domain could also bind to the double stranded DNA to regulate the expression of target genes (Liu, 2008; Caberoy et al., 2010). Taken together, the regulatory pathway of TLPs could be further refined.

Both TLPs and SKPs are the key components of the E3 ligase SCF complex in plants, which participates in selective protein degradation (Horn-Ghetko et al., 2021). In addition, both TLPs and SKPs have been proved to participate in seed germination, respectively (Hong et al., 2015; Wang et al., 2018; Li et al., 2021), thus we discussed whether TLPs and SKPs could interacted with each other to regulate the seed germination. We first analyzed the expression pattern of *TLPs* and *SKPs*. *Via* qRT-PCR, we focused on *OsTLP10*, *OsTLP12*, and *OSK20*. Next, using yeast two-hybrid assay and pull down, we confirmed the interaction between OsTLP10 and OSK20. In conclution, we showed that OsTLP10 played a key role in rice seed germination, and OsTLP10 and OSK20 that highly expressed in seeds interacted with each other. According to the phylogenetic tree, there were ten members in the same branch of OsTLP10, such as SiTLP13, SbTLP10, ZmTLP12, TaTLP32 and HvTLP8 (**Figure 5**). Most of their function has not been reported, thus our jobs provide the foundation for the functional exploration of these TLPs in future. Together, according to our genome-wide analysis of TLP gene family, we discussed the evolutionary relationship, expression levels, regulatory network and the interacting proteins of TLPs in 8 species, supplying the further research direction in different species.

## Conclusion

In this study, a comprehensive analysis of *TLP* genes in seven crops were performed, including *TLP* gene identification, phylogenetic analysis, classification, chromosomal distribution, gene duplication, conserved motifs, functional domains, gene structure, cis-elements and expression patterns of *TLP* genes. In addition, we confirmed that *OsTLP10* played a key role in rice seed germination, and OsTLP10 and OSK20 that highly expressed in seeds interacted with each other. Taken together, our results provide the foundation for the functional exploration of these TLPs in crops.

## Data availability statement

The original contributions presented in the study are included in the article/**Supplementary Material**. Further inquiries can be directed to the corresponding author.

## Author contributions

YZ and WH designed the study. YZ performed the entire experiment. JW performed the bioinformatic analysis. JF, HG performed vector construction. ZD, WZ and WX performed the RNA extraction. All authors contributed to the article and approved the submitted version.

## Supplementary material

Supplementary Figure 1 The sequence of conserved motifs in all TLP proteins

Supplementary Table 1 Basic information of *TLP* genes in all species which were analyzed in this work.

Supplementary Table 2 The protein sequences of OsTLP6, ZmTLP10 and ZmTLP15.

Supplementary Table 3 Protein sequences, theoretical pI and molecular weight of TLP family in all species.

Supplementary Table 4 Orthogroups in seven crops.

Supplementary Table 5 Ka/Ks ratios and divergence times of *TLP* gene pairs in same species.

Supplementary Table 6 Ka/Ks ratios of OsTLPs with TLPs from other species.

Supplementary Table 7 The expression values of *OsTLPs*, *ZmTLPs* and *TaTLPs* in multiple tissues.

Supplementary Table 8 The expression values of *OsTLPs* in different stresses.

Supplementary Table 9 Primers used in this article.
