# Supplementary figures and images for: Genome-wide identification and comprehensive analysis of tubby-like protein gene family in multiple crops
Source: Front Plant Sci. 2022 Dec 14;13:1093944. doi: 10.3389/fpls.2022.1093944 (PMC9795058; doi:10.3389/fpls.2022.1093944)

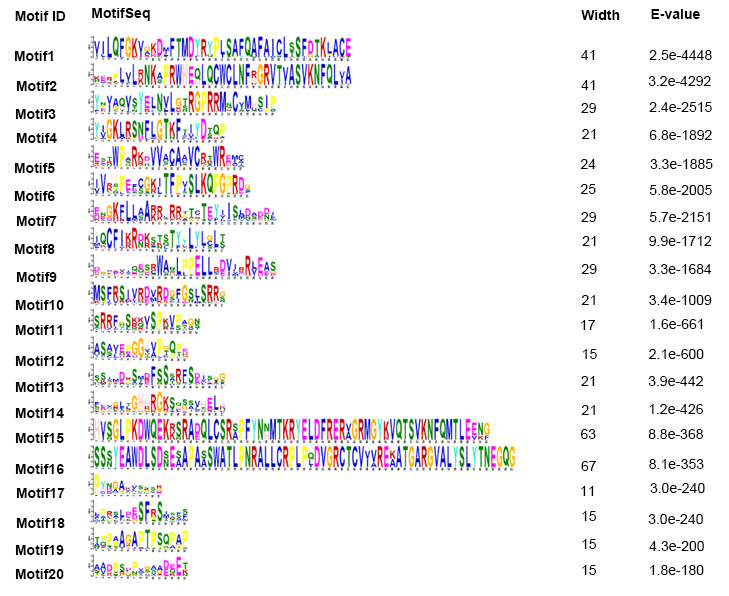

Supplement: Supplementary Figure 1 — The sequence of conserved motifs in all TLP proteins [file Image_1.jpeg]
